# Public opinion concerning residential sprinkler systems for 1- and 2-family homes

**DOI:** 10.1186/s40621-015-0060-5

**Published:** 2015-11-05

**Authors:** Shannon Frattaroli, Keshia M. Pollack, Phillip J. Cook, Michele Salomon, Elise Omaki, Andrea C. Gielen

**Affiliations:** 1 The Johns Hopkins Bloomberg School of Public Health, Center for Injury Research and Policy, 624 North Broadway, 5th Floor, Baltimore, MD 21205 USA; 2 Duke Sanford School of Public Policy, Durham, USA; 3 Harris Poll, Inc, New York, USA

**Keywords:** Residential fire sprinkler systems, Survey, Fire prevention

## Abstract

**Background:**

Residential sprinkler systems (RSS) are one intervention to prevent fire injury and death, yet there is no literature documenting why RSS homeowners opt to purchase a sprinkler-equipped home. This manuscript describes homeowners’ decisions to purchase homes with residential sprinkler systems (RSS) and their experiences with the technology. It also compares how RSS homeowners and owners of homes without RSS value sprinklers and their levels of support for policies to mandate RSS in new homes.

**Methods:**

We used a national online web panel to sample owners of 1- and 2-family homes, and descriptive methods to analyze the resulting data.

**Results:**

Our final sample included 1,357 homeowners of 1- and 2-family homes without RSS and 976 homeowners with RSS. RSS homeowners were more likely than owners of non-RSS homes to indicate they would buy an RSS home in the future (75 % vs. 30 %), and more often indicated a willingness to pay for sprinklers (70 % vs. 40 %). RSS homeowners also expressed higher levels of support for policies to mandate RSS in all new 1- and 2-family homes (48 % vs. 19 %).

**Conclusions:**

The findings offer insight into educational and policy strategies to promote RSS in all new homes, and provide a foundation for future research in this area.

## Background

Between 2000 and 2014 42,370 people died in residential fires in the United States (U.S.) (Hylton JGH 2015). These deaths, and the injuries sustained by others in house fires have long been viewed as a preventable public health problem. In 1735 Benjamin Franklin, founder of the modern fire service, famously wrote about house fires, “an ounce of prevention is worth a pound of cure” (Franklin 1735). By the 1900s, fire prevention strategies had moved beyond Franklin’s admonitions “to take care how they suffer living coals in a full shovel” (Franklin 1735) to include passive technologies that suppress fire. Perhaps this evolution is best reflected in the 1973 publication, *America Burning: The Report of the National Commission on Fire Prevention and Control,* which provided the first national assessment of the U.S. fire problem and identified strategies for reducing fire-related deaths (National Commission on Fire Prevention and Control 1973). With attention to education, smoke alarms, and further development of “automatic extinguishing systems” the Commission operationalized fire prevention for the nation and updated Franklin’s notion of prevention for the modern era. While forward progress on all of these strategies has occurred in the decades since the report (Kendrick et al. 2012; Warda and Ballesteros 2007), and fire deaths have declined (Karter 2014), there is more work to be done (Recommissioned Panel for Burning Recommissioned Panel for America Burning 2002).

Arguably, the intervention for which there is the most to be done in terms of education, policy change, social norms, and uptake of the technology is residential sprinkler systems (RSS). Sprinkler systems have been used to protect commercial property for more than a century, and the technology for residential applications has existed for decades (Coleman 1985). The available evidence suggests that when fires occur in sprinkler-equipped homes, there are fewer deaths and injuries, and less property damage compared to homes without sprinklers (Weatherby 2009; Ford 1997; Hall 2013). A 2012 article affirms the life and property savings, and concludes that RSS are also cost-effective (Butry 2012). The available evidence demonstrates the effectiveness of RSS in reducing fire-related death, injury and property loss.

Since the 1980s, model building codes, which offer states and localities a uniform set of standards that they may choose to adopt but have no force of law unless enacted into law, have mandated RSS in all new multifamily residences (Wieczorek and Perdue 2011). With the 2009 update of the International Residential Code (IRC) to include RSS in all new 1- and 2-family homes, the model codes administered by the National Fire Protection Association and the International Code Council now mandate sprinklers in all new residences (Wieczorek and Perdue 2011). As of this writing, California, Maryland and the District of Columbia have adopted the model codes and require sprinklers to be installed in all new 1- and 2- family homes. The three decades between the code mandate for multifamily residences and 1- and 2-family homes and the subsequent adoption of those model codes by states and localities likely explain the difference in RSS prevalence among housing types. An estimated 2 % of 1- and 2- family homes are sprinkler-equipped compared to 13 % of homes in multi-unit buildings and 32 % of homes in buildings with more than 49 units (Hall 2013).

The 2009 code change followed decades of advocacy by fire prevention professionals that yielded over four hundred local ordinances requiring RSS in new 1- and 2-family homes (Pertschuk et al. 2013; Frattaroli et al. 2013; Fire Sprinkler Initiative 2014). However, adoption of the 2009 IRC by states has been mixed, with opponents most often expressing concerns about the cost impact of an RSS mandate and the potential for the added costs to negatively impact the new housing market. As of December 2014, in addition to the 2 states (California and Maryland) and the District of Columbia that require RSS in all new 1- and 2-family homes, 14 states have new policies prohibiting adoption of the IRC RSS mandate (Fire Sprinkler Initiative 2014). Advocacy efforts to promote RSS policy expansion have proceeded largely without homeowners. A national poll conducted in 2005 revealed that 45 % of homeowners believed a sprinkler-equipped home is more appealing than a home without sprinklers; 69 % believed RSS increases a home’s value; and 38 % indicated they would be more likely to buy a home with sprinklers than one without (Home Fire Sprinkler Coalition 2015) While this survey generated important information, it did not capture the experiences of people who own sprinkler-equipped homes, the value they place on this technology, or their support for policies that mandate RSS in new homes. We designed and fielded a survey to address these knowledge gaps.

The aims of this paper are to: 1) describe socio-demographic correlates of living in homes with RSS; 2) describe the decisions of homeowners to purchase RSS homes and their experiences with RSS; and 3) compare how RSS homeowners and owners of homes without RSS value sprinklers, and their levels of support for policies mandating RSS. These findings inform RSS education and policy advocacy efforts, and provide a foundation for future, hypothesis-driven research about this topic.

## Methods

We sought a sampling strategy to learn about experiences with sprinklers from a large, diverse sample. Harris Poll maintains the Harris Poll Online, an extensive worldwide panel of respondents who enroll to participate in survey research. The panel has been used to field surveys on several public health topics (Pollack et al. 2010; Pignone et al. 2007; Klein et al. 2007). For this study, it created an efficient way of accessing hard-to-identify homeowners that had experience with RSS.

### Sample

We targeted web panel members who were U.S. owners of 1- and 2-family homes at least 18 years of age. Each week from August 16 to September 24, 2012, the Harris team identified a random sample of participants meeting the selection criteria. Approximately 385,000 members received an invitation to respond to the survey. Those who did not respond received one follow-up invitation. We oversampled individuals classified as Black/African American or Hispanic in order to assure sufficient participation from these groups to allow for comparisons by race and ethnicity. Oversamples can compensate for possible lower response rates and, in the case of this study specifically, allowed for each racial and ethnic group to be demographically representative as well. The total population, as well as Black/African American and Hispanic responders, were weighted separately and then post-weighted into a representative total. To have a sufficient number of respondents to inform our comparisons between RSS homeowners and owners of homes without sprinklers, we aimed to collect responses from 1,000 respondents from sprinkler-equipped homes and an additional 1,000 respondents who were owners of homes without RSS.

### Instrument development

We first identified areas of interest (RSS in the home, decision to purchase an RSS-equipped home, experience with current RSS, other injury prevention devices in the home, attitudes and beliefs about fire prevention, RSS in future homes and the value of RSS, and home fire experience in the community) and then developed questions for each area. To assess the value homeowners place on RSS, we used a willingness to pay approach. Specifically, we constructed questions to present those who reported the square footage of their homes with a dollar figure based on a national average cost per square foot ($1.61) of installing an RSS in a new home the same size as their current home (Newport Partners 2008).

RSS experts reviewed the draft survey for content; the Harris team reviewed the draft for readability and design considerations. We piloted the initial online version among several members of the research team and with colleagues unfamiliar with this topic. The fielded version incorporated feedback from each of these stages.

There were two versions of the survey: one for owners of sprinkler-equipped homes and a second, shorter version for those living in homes without RSS. Two screening questions determined eligibility for the survey: did the respondent own their home and was it a 1- or 2-family home? A third question (Does your home have an indoor home fire sprinkler system designed to turn on if there is a fire in your home?) assessed which version of the survey the homeowner would be eligible to complete.

### Data collection

To access the survey, invited panel members logged onto the Harris site. The system assures only invited members have access to the survey and guards against any one person completing the survey multiple times. Members who completed the survey received points in the Harris system that can be redeemed for products and services. On average, respondents completed the survey in 14 min.

### Data analysis

The Harris team weighted the data to be representative of the U.S. population of homeowners 18 years and older. Each category of the sample (owners of RSS homes, owners of non-RSS homes, general population, Black/African American oversample, Hispanic oversample) was weighted using the 2011 Current Population Survey (http://www.census.gov/cps/methodology/) by the following key demographic variables: household income, education, age, gender, and region. Even though the pool of respondents was not obtained through a probability sample, it is demographically consistent with U.S. homeowners. Harris sampling and weighting procedures included a propensity score to account for the potential biases related to attitudes and behaviors that can arise when using an online panel. This addressed the biases beyond those corrected through weighting based on demographic characteristics alone. Through this process, potential biases related to being active online, joining a panel, and responding to particular surveys, were minimized, thus further enhancing the sample’s representativeness (Pollack et al. 2010).

Descriptive statistics of respondent characteristics are presented as weighted proportions. Bivariate relationships of responses between RSS homeowners and owners of homes without sprinklers were analyzed using t-tests of proportions with Quantum, SPSS, a commonly used software program designed specifically for market research analysis that provides the ability for survey data to be easily tabulated and analyzed, including tests for statistical significance (Quantum 1997). Data are presented for all homeowners, and separately for homeowners with and without RSS. Statistical significance was established at *p* < 0.05. The Harris team reviewed and coded open-ended responses that inform the study aims.

The Johns Hopkins Bloomberg School of Public Health Institutional Review Board reviewed this research and deemed it exempt.

## Results

Of the approximately 385,000 invitations sent to panel members, 50,526 responded; more than half (57 %) did not meet one or more of the eligibility criteria. A total of 5,433 members were eligible to participate and responded. Of that number, 3,100 began the survey but did not complete it. An additional 16,404 members responded, but their responses exceeded the quota. The final sample included 1,357 homeowners of 1- and 2-family homes without RSS, and 976 homeowners of 1- and 2- family homes that were equipped with RSS at the time they completed the survey. Further results are presented as weighted percentages.

### Comparison between RSS and non-RSS home occupants

RSS homeowners lived in newer and larger homes, reported higher incomes, higher educational achievement, and more disabilities than those living in homes without sprinkler systems. (Table 1) They also reported higher compliance with two of three home safety measures assessed: having a smoke alarm (97 % vs. 94 %) and a carbon monoxide alarm (62 % vs. 56 %).

**Table 1:** Respondent demographics

	RSS	Non-RSS	
	(*n* = 976)	(*n* = 1357)	
Gender (male)	49 %	49 %	*p* = NS
Age (mean years)	45.0	53.6	*p* < 0.05
Education			
High school (less, some, completed)	17 %	31 %	*p* < 0.05
College (some, degree)	55 %	50 %	*p* < 0.05
Graduate school (some, degree)	25 %	17 %	*p* < 0.05
Job training after HS	2 %	2 %	*p* = NS
Race/Ethnicity			
White	65 %	79 %	*p* < 0.05
Black	13 %	9 %	*p* < 0.05
Asian/Pacific Islander	6 %	2 %	*p* < 0.05
Native American	1 %	1 %	*p* = NS
Hispanic	12 %	7 %	*p* = NS
Other/Decline	3 %	2 %	*p* = NS
Employment			
Employed full-time	56 %	47 %	*p* = NS
Employed part-time	11 %	14 %	*p* = NS
Self-employed	4 %	4 %	*p* = NS
Not employed, looking for work	5 %	3 %	*p* = NS
Not employed, not looking for work	2 %	4 %	*p* = NS
Retired	14 %	23 %	*p* < 0.05
Not employed, disability or illness	2 %	2 %	*p* = NS
Student	2 %	2 %	*p* = NS
Stay-at-home spouse or partner	5 %	2 %	*p* < 0.05
Household Income			
<$35,000	13 %	18 %	*p* < 0.05
$35,000-$49,999	10 %	12 %	*p* = NS
$50,000-$74,999	18 %	21 %	*p* = NS
$75,000-$99,999	17 %	17 %	*p* = NS
$100,000-$124,999	11 %	11 %	*p* = NS
$125,000-$149,999	9 %	7 %	*p* = NS
$150,000-$199,999	6 %	5 %	*p* = NS
$200,000-$249,999	3 %	2 %	*p* = NS
>$250,000	6 %	1 %	*p* < 0.05
Decline to answer	8 %	5 %	*p* < 0.05
Someone in the Home has a Disability			
Blind/severe visual impairment	18 %	6 %	*p* < 0.05
Deaf or hard of hearing	27 %	24 %	*p* = NS
Long-lasting condition that limits basic physical activity	28 %	20 %	*p* < 0.05
Long-lasting condition that makes learning, remembering, concentrating difficult	20 %	7 %	*p* < 0.05
Neighborhood			
Urban	29 %	19 %	*p* < 0.05
Suburban	48 %	42 %	*p* < 0.05
Rural	21 %	39 %	*p* < 0.05
Square footage of house			
<1,000	2 %	4 %	*p* < 0.05
1,001-2,000	23 %	47 %	*p* < 0.05
2,001-3,000	34 %	30 %	*p* = NS
3,001-4,000	19 %	9 %	*p* < 0.05
4,001-5,000	8 %	3 %	*p* < 0.05
>5,000	8 %	1 %	*p* < 0.05
Not sure	7 %	7 %	*p* = NS
Year moved in	2002	1994	*p* < 0.001
Year house built	1990	1970	*p* < 0.001
Working smoke alarm (yes)	94 %	97 %	*p* < 0.05
Working CO detector (yes)	62 %	56 %	*p* < 0.05
Emergency Exit Plan (yes)	78 %	76 %	*p* = NS

### Decision to purchase an RSS home

Of the RSS homeowners (*N* = 976), most (80 %) reported their home was sprinkler-equipped when they moved in, with the majority of this group (62 %) having bought homes with an RSS in place, and more than one-third either having chosen sprinklers as an option offered by their builder (19 %) or having had sprinklers installed prior to moving in (15 %). The remainder (20 %) of RSS homeowners reported retrofitting their home with an RSS after moving in, with 87 % reporting that this occurred during a home improvement project. Homeowners who reported retrofitting their homes with sprinkler systems often did so by choice: only 11 % indicated they installed their sprinklers to comply with a local policy mandate that required RSS as part of a substantial renovation.

Homeowners reported learning about RSS from a variety of sources (Table 2), and these sources were generally helpful to them. When asked to rate their sources on a scale of 1 to 5 (with 5 being the most helpful), the lowest mean score was 3.7 and applied to three groups (someone who lived in an RSS house, friends or family, and real estate agent) and fire service representatives rated highest (4.2). While these groups were important sources of information about RSS, between 12-15 % of homeowners reported that someone from each of those groups recommended against buying a sprinkler-equipped home. Sixteen percent of homeowners indicated they did not know about RSS when they purchased their current sprinkler-equipped homes.

**Table 2:** Sources of information about residential sprinkler systems (RSS) among homeowners living in sprinkler-equipped homes

	Learned about RSS from (*n* = 976)	Helpful in learning about RSS^a^	Recommended buying RSS^b^ (*N* = 667)
Friend/family	24 %	3.7	19 %
Builder	23 %	3.9	16 %
Real estate agent	19 %	3.7	13 %
Insurance agent	19 %	4.0	16 %
Fire service representative	15 %	4.2	14 %
Someone who lived in a sprinkler-equipped home	18 %	3.7	14 %
Media	13 %	N/A	N/A
I had lived in a sprinkler-equipped home	21 %	N/A	N/A
I did not know about RSS when I was looking to purchase my home	16 %	N/A	N/A
Other	3 %	N/A	N/A

^a^Mean score on a scale of 1–5, where 5 is extremely helpful and 1 is not helpful at all

^b^All respondents who answered they had learned about RSS from one or more of these groups were asked, “Did any of the following people (you learned about RSS from) actually recommend that you purchase a house with a home fire sprinkler system?”

Among the homeowners who purchased homes with sprinklers already installed (*n* = 785), more than half (52 %) reported that the RSS made them more likely to purchase the home. When asked an open-ended question about why they were more likely, safety considerations were most cited (53 %) followed by financial incentives (11 %), although 31 % provided no reason. The findings provide less insight into why 11 % reported that the RSS made them less likely to purchase since more than half of this group opted not to include a text response (52 %), or responded “nothing” or “don’t know” (17 %). Of the 72 homeowners who provided an explanation, cost (9 %), safety (9 %), and concerns about the sprinklers activating spontaneously (4 %) or breaking (3 %) were most common.

### Living in an RSS home

Among respondents living in a sprinkler-equipped home (*n* = 976), 10 % reported having had a fire in their current home that prompted a fire department response. Among these fires, 74 % triggered smoke alarms. Of the fires that triggered smoke alarms (unweighted *n* = 69), 73 % also activated the sprinkler system. We have no data as to whether the RSS activated for the 26 % of fires that did not trigger the smoke alarms. When activated, sprinklers “put out the fire completely” in slightly more than half of the fires (52 %) and in 42 % the RSS controlled the fire but did not extinguish it before the fire department arrived. The remaining homeowners (6 %) were unsure about the extent to which the RSS controlled the fire. Of the 14 fires that activated the smoke alarm, but not the RSS, the large majority reported the fires were too small to activate the sprinklers (unweighted *n* = 7) or occurred in homes where the RSS had been turned off (unweighted *n* = 3). A minority of homeowners reported that the RSS was on but did not work (unweighted *n* = 2), or that they were unsure why the sprinklers did not activate (unweighted *n* = 2).

Of all RSS homeowners (n = 976) 9 % reported a sprinkler activation due to a fire that did not involve a fire department response. These activations extinguished the fires, sometimes in combination with another suppression method such as a handheld fire extinguisher or a blanket.

Eleven percent of all RSS homeowners reported sprinkler activations not caused by fire. Upon examination of the open text responses detailing the circumstances of the non-fire activations (*n* = 103), the largest category other than “decline to answer/don’t know/none” (53 %) was activations coincident with smoke and/or fire (30 %), suggesting the RSS was activated appropriately and that some respondents may have interpreted the question differently from what was intended. These text responses also included malfunction (5 %), electrical short (1 %), and 4 % who activated their RSS intentionally to verify they worked, in addition to 8 % who provided miscellaneous responses. One percent of all RSS respondents (*n* = 976) reported sprinkler activations due to system malfunction or an electrical short.

### Value of RSS to homeowners

Owners of sprinkler-equipped homes were more likely than owners of homes without sprinklers to agree the benefits outweigh the costs (77 % vs. 46 %), while those in homes without RSS were more likely to agree the costs outweigh the benefits (54 % vs. 23 %). (Table 3) When asked if they would choose a sprinkler-equipped home in the future, 75 % of those living in an RSS home vs. 30 % of those living in a home without sprinklers said they would. When asked if they would be willing to pay a specific amount for a sprinkler system in a new house the size of their current home, RSS homeowners more often indicated they would pay for sprinklers compared to homeowners living in non-RSS homes (70 % vs. 40 %). Those living in homes without sprinklers relative to those living in RSS homes reported higher rates of uncertainty about whether they would choose a sprinkler-equipped home in the future and their willingness to pay for RSS.

**Table 3:** Homeowners’ assessments of residential sprinkler systems (RSS)

	RSS	Non-RSS	
*When evaluating the benefits vs. costs of having a home fire sprinkler system, which of the following comes closest to your thoughts on the issue?*	*N* = 976	*N* = 1357	
Benefits strongly outweigh the costs	46 %	12 %	*p* < 0.05
Benefits somewhat outweigh the costs	31 %	34 %	*p* = NS
Costs somewhat outweigh the benefits	12 %	37 %	*p* < 0.05
Costs strongly outweigh the benefits	11 %	17 %	*p* < 0.05
*Now imagine you decided to move and buy or build a new home. Would you want a home fire sprinkler system in that house?*	*N* = 976	*N* = 1357	
Yes	75 %	30 %	*p* < 0.05
No	5 %	17 %	*p* < 0.05
Not Sure	20 %	53 %	*p* < 0.05
*Would you be willing to pay the increased amount [dollar amount provided based on square footage of current home] for a home fire sprinkler system in a new house the size of your current home?*	*N* = 921	*N* = 1254	
Yes	70 %	40 %	*p* < 0.05
No	12 %	28 %	*p* < 0.05
Not Sure	18 %	33 %	*p* < 0.05
*Do you support or oppose local laws that require new homes to have home fire sprinkler systems?*	*N* = 976	*N* = 1357	
Very much support	27 %	4 %	*p* < 0.05
Somewhat support	21 %	15 %	*p* < 0.05
Neither Support nor Oppose	33 %	46 %	*p* < 0.05
Somewhat oppose	10 %	18 %	*p* < 0.05
Very much oppose	8 %	16 %	*p* < 0.05
Mean score, with 1 = very much oppose and 5 = very much support	3.5	2.7	*p* < 0.05

### Support for RSS policies

Homeowners living in sprinkler-equipped homes were more than twice as likely as those in homes without RSS to support mandatory sprinkler laws for new 1- and 2-family homes (48 % vs. 19 %, Table 3). One-third of homeowners in sprinkler-equipped homes and 46 % of those in non-RSS homes were neutral, while 18 % and 34 % of the respective groups were opposed. Across the entire sample, the most common reason selected for supporting such a mandate was that “lives will be saved” (88 %) followed by “the fire service is supportive” of such laws (40 %). Opponents of such laws most often selected “homeowners should be able to choose whether to live in a home with a fire sprinkler system” (86 %) followed by concerns about cost (48 %) as their reasons for opposing RSS mandates.

## Discussion

These survey findings provide the first national exploration of how owners of 1- and 2-family sprinkler-equipped homes came to live in those homes; their experiences with the technology; their willingness to pay for RSS; and their support for policies to mandate sprinkler systems in all new 1- and 2-family homes. An earlier survey provides insight into the population of U.S. homeowners on these matters (Home Fire Sprinkler Coalition 2015), but the questions and sample are sufficiently different to preclude comparison. The current findings provide important new information about how people view and value RSS and policies to promote their inclusion in new homes. These results offer guidance for advocates, policymakers, practitioners, and community members about the role of RSS in future fire prevention strategies.

Owners of RSS-equipped homes are different from the general population of homeowners: they have higher incomes; live in larger, newer homes; report higher compliance with selected home safety behaviors; and are more likely to report that someone in the home has a disability. The finding that large proportions of those living in sprinkler-equipped homes chose to install sprinklers (54 %) and would choose them again (75 %) suggests there are people who will accept the technology when it is in place, choose the technology when it is offered, and seek it out when it is not already available. Moreover, when asked about future home buying, most homeowners were either supportive or uncertain about buying a home with RSS technology (95 % among those living in RSS homes and 83 % among those in homes without RSS). This support declined slightly when provided with information about the additional cost of an RSS (88 % among RSS homeowners; 73 % among owners of non-RSS homes). This lack of strong opposition to RSS presents an opportunity for educational and policy efforts to increase RSS in new homes, and to affect more general social norms on this issue.

### Promoting RSS through education

Efforts to raise awareness about RSS within housing-related professional groups are needed. Most homeowners we surveyed reported they learned about RSS from members of the fire service, realtors, builders, and the insurance industry. While most indicated that professionals in each of these groups were helpful when asked about RSS, there were also reports to the contrary. Efforts to assure that real estate and insurance agents are knowledgeable about RSS are needed. Tailored educational materials for these professionals are currently available from fire safety organizations (Fire Sprinkler Initiative 2014; Home Fire Sprinkler Coalition 2013, 2015; Newport Partners 2008). Incorporating RSS into professional training and certification or licensure procedures, and raising current expectations for RSS knowledge are additional strategies for assuring that the people who are interacting with homebuyers (e.g., real estate agents) are informed and informative about RSS. Research to understand the impact and reach of such strategies, particularly as they relate to the fire service, and how to maximize their impact is needed.

Friends and family were the most often cited resource for information about RSS among those living in sprinkler-equipped homes. They were the most frequently cited group to recommend buying a sprinkler-equipped home to RSS homeowners in our sample, but among the lowest ranked groups in terms of helpfulness. Furthermore, 16 % of RSS homeowners reported not knowing about RSS when they purchased their homes. Effective methods for disseminating information about RSS to the public are needed.

While the purchase of a home provides an opportunity to consider RSS, our data indicate that retrofitting is the avenue by which a sizeable portion (20 %) of 1- and 2-family homes became sprinkler-equipped. Efforts to educate homeowners and contractors about retrofit options and the benefits of RSS are needed to assure that sprinklers are considered when planning a home remodel. We are unaware of any large-scale effort to promote RSS retrofit among homeowners or contractors.

### RSS and the housing market

Those living in sprinkler-equipped homes report positive (extinguished a fire) and negative (system malfunctioned) experiences, although the negative experiences represent one percent of RSS homeowners in the survey. The predominance of positive experiences likely explains why most (75 %) participants report wanting sprinklers in future homes and being willing to pay for them (70 %). This endorsement from RSS users suggests that familiarity with the technology may be one factor in building a loyal customer base to support RSS and realize a safer housing stock. Fully 52 % of homeowners who purchased RSS homes reported the sprinkler systems made them more likely to buy their home, with the majority (53 %) citing safety benefits as the reason for their interest in RSS. These safety benefits may provide an additional point to convey to homebuyers, builders, and real estate agents.

### Promoting RSS through Policy

Our findings show that about 22 % of all homeowners and nearly half of those in RSS homes, support mandates to assure new 1- and 2- family homes are sprinkler-equipped; 45 % (the largest percentage) of all homeowners were undecided. Among supporters, the strength of the safety argument offers further support for the idea that safety features are important to homeowners, and may be influential in generating support for policies to mandate RSS in 1- and 2-family homes. Given the higher proportion of people with disabilities living in RSS-equipped homes in our sample, emphasizing the benefits that RSS may provide to people who have more difficulty exiting a home quickly is another aspect of the safety argument that may appeal to people who are undecided about RSS.

Support for public health policy interventions must be balanced against individual freedoms. For many opponents of residential sprinkler mandates (34 % of our total sample), whether to live in a sprinkler-equipped house should be a choice. Presenting the sprinkler mandate as part of a larger system that governs residential construction to set minimum safety standards may help opponents to understand this policy as part of a larger set of policies to improve home safety for residents.

### Limitations

Our data are based on self-reported information collected through an online survey. Therefore, we have no way to verify responses. The inability to validate responses is compounded by the fact that fires and sprinkler activations are rare events that yielded small numbers in our final sample. With our large sample size of 976 RSS-equipped homeowners, we were well powered to detect these rare events. Despite these limitations, these findings present an important contribution because of the dearth of information on this topic. There is a need for additional research to better understand the prevalence and circumstances of fires extinguished and controlled by sprinklers, and the resulting benefits, as well as failures of the technology and the consequences of those failures.

Our findings reflect a non-random sample. Because our survey sought owners of 1- and 2-family sprinkler-equipped homes, a population that represents a small minority of homeowners (2 %) and for which there is no registry or known method to access them, a random sample proved prohibitive from a resource standpoint. Our sampling strategy proved efficient and effective, providing the first nationally representative data from U.S. 1- and 2-family adult homeowners and previously undocumented information from a portion of the population affected by current RSS policy discussions.

Many of our results point to comparisons between homeowners living in sprinkler-equipped homes and those living in homes without RSS. Our cross-sectional data do not reveal the extent to which differences between the two populations are directly related to the homeowners’ decision to live in a sprinkler-equipped home, or whether and how those differences can be addressed to effectively assure greater access to RSS.

## Conclusion

These results provide the first nationally representative data from owners of 1- and 2-family sprinkler-equipped homes. The findings are relevant to the current policy discussion about whether states and localities should adopt the model codes that require RSS in new 1- and 2-family homes. They also have implications for education and advocacy strategies and point to the need to raise awareness about the value of RSS among homeowners and future homeowners.
